# Nurses’ Guide to Surgical Bandaging and Dressing

**Published:** 1903-12

**Authors:** 


Nurses' Guide to Surgical Bandaging and Dressing. By William Johnson Smith, F. R. C. S., Principal Medical Officer, Seamen's Hospital, Greenwich. Philadelphia: J. B. Lippincott Co. 1903. (Price, 75 cents.)

This is a book written for the instruction of pupil nurses, particularly those in surgical wards. We commend it to hospital superintendents and others interested in the training of nurses. It is well adapted for the purpose named and also is a valuable addition to the literature for the advanced nurse. M. J. F.

